# Recovery‐Oriented Care Planning in Mental Healthcare Services: A Combined Concept Analysis and Scoping Review

**DOI:** 10.1111/inm.70337

**Published:** 2026-08-19

**Authors:** Natthapon Inta, Prapan Phetlerthirunkul, Jirada Kaewchuchuen, Chonmanan Khanthavudh, Wilailak Sakharang

**Affiliations:** ^1^ Princess Agrarajakumari Faculty of Nursing Chulabhorn Royal Academy Bangkok Thailand; ^2^ Florence Nightingale Faculty of Nursing, Midwifery & Palliative Care King's College London London UK; ^3^ Faculty of Nursing Srinakharinwirot University Nakhon Nayok Thailand; ^4^ Ramathibodi School of Nursing, Faculty of Medicine Ramathibodi Hospital Mahidol University Bangkok Thailand; ^5^ Cicely Saunders Institute of Palliative Care, Policy and Rehabilitation, Florence Nightingale Faculty of Nursing, Midwifery and Palliative Care King's College London London UK

**Keywords:** care planning, concept analysis, mental health, personal recovery, recovery‐oriented practice

## Abstract

Recovery‐oriented care planning is increasingly promoted within international mental health policy and practice; however, the concept remains inconsistently defined and operationalised across the literature. This study aimed to clarify the concept of recovery‐oriented care planning using Walker and Avant's concept analysis method alongside a scoping review. Literature was systematically searched across six electronic databases: MEDLINE, EMBASE, PsycINFO, CINAHL, ProQuest One Academic and Scopus. Data were analysed across 28 included papers to identify antecedents, defining attributes, consequences, empirical referents and model cases of recovery‐oriented care planning. The findings demonstrated that recovery‐oriented care planning emerged from broader philosophical and policy shifts away from paternalistic and symptom‐focused models towards collaborative, person‐centred and recovery‐oriented mental healthcare. Defining attributes included collaboration and shared decision‐making, strengths‐based and person‐centred approaches, recovery‐focused goal setting, empowering therapeutic relationships and viewing care planning as an ongoing and dynamic process. Recovery‐oriented care planning was associated with increased empowerment, hope, autonomy, therapeutic alliance, self‐management and more holistic and individualised care. Empirical referents included recovery‐oriented care planning tools, collaborative planning processes and recovery‐focused outcome and fidelity measures. This concept analysis provides greater conceptual clarity regarding recovery‐oriented care planning and may support future research, education, policy development and implementation of recovery‐oriented mental healthcare practices.

## 1 Background

Since the second half of the 20th century, mental healthcare systems across Western and other high‐income countries have undergone substantial conceptual and structural transformation (Rössler and Drake 2017), during which the concept of mental health recovery has become an increasingly prominent topic of discussion within mental health settings (Jacob et al. 2017). This paradigmatic shift moved the focus away from clinical recovery centred on psychopathology and dysfunction towards personal recovery, which emphasises living a meaningful and satisfying life alongside achieving optimal functioning (Yüksel et al. 2025). Care planning represents one approach through which these recovery principles can be translated from conceptual understanding into everyday clinical practice.

Care planning is an essential process in mental health care that supports services to respond to a person's changing needs by setting out goals, actions, and the type, frequency and level of support required to guide treatment (Damant et al. 2026; Reid et al. 2018). The process of care planning involves exploring and agreeing upon the individual's strengths and needs, followed by developing a written care plan that is implemented and regularly reviewed to monitor the individual's progress (Kemp et al. 2024). Care plans can serve multiple roles, including acting as an extension of the medical record, a guide for care delivery and an agreement between individuals with mental health conditions, their healthcare providers and the wider health system (Burt et al. 2014). In this way, they may be used to document additional important information for the ongoing management of long‐term conditions. Consequently, the use of care planning and written care plans has been suggested as a way to improve the management of long‐term conditions (Reeves et al. 2014), including mental health conditions.

In practice, the content of care plans has traditionally been largely determined by service providers, reflecting a predominant focus on clinical recovery. Although care plans can be developed by various health professionals, nursing care plans represent one of the most explicit and widely recognised examples of care planning in practice. Within the nursing profession, the nursing process, introduced in 1958, provides a structured framework for care planning and care delivery (Toney‐Butler and Thayer 2026), comprising six stages: assessment, diagnosis, outcome identification, planning, implementation and evaluation (Open Resources for Nursing 2021). Nursing diagnoses developed by the North American Nursing Diagnosis Association (NANDA) are widely used internationally, including in European countries (Cristina Dos Santos et al. 2024; Fumai 2025), Korea (Shin et al. 2021), Thailand (Takahiro et al. 2025) and Nigeria (Enebeli et al. 2023), because they provide standardised terminology that supports consistent communication, clinical judgement and the measurement of nursing care across the professions (Mueller Staub 2022; Rodríguez‐Suárez et al. 2025; Toney‐Butler and Thayer 2026). However, this process often reflects a traditional biomedical approach, in which care planning is guided by deficit and illness‐focused models that emphasise impairments and rely primarily on professional judgement to determine individuals' needs (Tondora et al. 2014). Consequently, care plans within mental health services, particularly in acute settings, are often developed with limited or no involvement from service users (Reid et al. 2018), restricting the extent to which their views are incorporated and raising concerns that control and containment may take precedence over treatment and support (Moyo et al. 2022).

In line with the promotion of mental health recovery, which emphasises supporting people with mental health challenges to find purpose and meaning in their lives through individualised and value‐informed care, active involvement of individuals in their own care is prioritised (Anthony 1993; Berger 2006; Strand et al. 2017). This aligns with the National Institute for Health and Care Excellence (NICE) guideline of rehabilitation for adults with complex psychosis, which recommends that care plans should be developed collaboratively with the person, address identified needs including both physical and mental health, incorporate the person's recovery goals, and clearly outline actions and responsibilities for staff, the individual and their family or carers (National Institute for Health Care Excellence 2020). Similarly, the Care Programme Approach (CPA) was introduced in England and Wales (United Kingdom) as a framework to provide a more structured and consistent way for local services to support people with severe mental health conditions in the community, through care coordination, care planning and case management (Kingdon 1994). The framework outlined requirements for comprehensive health and social care assessments, including risk assessment and promoted the active involvement of service users, carers, multidisciplinary teams and external agencies in care planning and delivery, based on the principle that all individuals receiving mental health services should have a key worker, care plan and regular review, with the level of professional involvement varying according to the complexity of need (Kingdon 2019). Although the CPA has been revised several times and the most recent versions are explicitly recovery‐oriented, its emphasis appeared to remain on ensuring that service users stayed engaged with services and received coordinated, collaborative support.

To translate recovery‐oriented principles into care planning processes within clinical practice, care plan templates have been widely introduced to promote person‐centred and collaborative approaches to supporting recovery (Inta, Grealish, et al. 2026; Inta, Leamy, et al. 2026; Kemp et al. 2024). However, there remains limited clarity regarding what recovery‐oriented care planning actually means, how it should be implemented in practice and the extent to which it is consistently enacted in real‐world settings. Previous research has largely focused on the development of recovery‐oriented practice guidance, whereas this study extends existing knowledge by examining how recovery‐oriented principles are operationalised within care planning at the practical and clinical level, thereby addressing an important gap in understanding the implementation of recovery‐oriented care planning in practice.

## 2 The Review

### 2.1 Aims

This study aimed to analyse and clarify the concept of mental health recovery‐oriented care planning by identifying and clarifying its definition, attributes (key features or concepts), antecedents (the underlying factors for its emergence, its history and evolution) and consequences (benefits and impacts of its utilisation) in mental health‐related practice and research.

### 2.2 Research Questions

The review also addressed two main research questions which were as follows:

1. How is the recovery‐oriented care planning defined or conceptualised in mental health practice and research?
2. What are the key antecedents, attributes and consequences of mental health recovery‐oriented care planning?

## 3 Methods

This concept analysis was conducted in line with eight steps of Walker and Avant's method of concept analysis (Walker and Avant 2005): (1) choosing a concept, (2) determining the aims of the analysis, (3) identifying the uses of the concept, (4) determining the defining attributes, (5) identifying the model case and additional cases, (6) identifying antecedents, (7) identifying consequences, (8) defining empirical referents. This method helps to clarify the concept by making its defining characteristics more explicit. A model case is constructed as a “real life” example illustrating all key attributes of the concept (Walker and Avant 2005). Additional cases, including borderline, related and contrary examples, allow variations exploration in the concept, refine its boundaries and distinguish it from closely related ideas (Walker and Avant 2005). Identified antecedents and consequences also provide further explanation of the conditions under which the concept occurs and its potential outcomes (Walker and Avant 2005). Finally, empirical referents, or observable indicators, are established to ensure the concept's practical applicability and measurability (Walker and Avant 2005).

In addition to concept analysis, we utilised a scoping review approach to further strengthen a more systematic and structured literature search, undertaken as an integral method to enhance transparency and reproducibility throughout the process (Lam Wai Shun et al. 2022). The scoping review followed the JBI methodology and aimed to identify and examine how recovery‐oriented care planning has been used and defined in the existing literature (Peters et al. 2022). The review included the following stages: (1) search strategy, (2) study or source of evidence selection, (3) data extraction and (4) data analysis and presentation (Peters et al. 2022). The study was reported in line with the PRISMA Extension for Scoping Reviews (PRISMA‐ScR) checklist (Tricco et al. 2018).

### 3.1 Search Strategy

A systematic search was conducted using free text and indexed terms, including truncation (*), proximity operators, and MeSH terms or subject headings, which were modified for each database. To ensure a comprehensive search capturing all relevant elements, three key concepts aligned with PCC (Population, Concept, Context) framework (Peters et al. 2020) were used to develop the search strategy: Population (mental health) AND Concept (recovery OR recovery orient*) AND Context (care plan* process). The search was completed in January 2025 and updated in May 2026 across six databases: MEDLINE, EMBASE, PsycINFO, CINAHL, ProQuest One Academic and Scopus. No restrictions were applied regarding publication date, language, or country of origin. All studies published from database inception up to the date of the final search were considered for inclusion in screening process.

### 3.2 Study and Source of Evidence Selection

Studies were included if they: (1) focused on recovery‐oriented care planning used by healthcare professionals for people experiencing mental health conditions; (2) were published as empirical studies of any design, literature reviews, meta analyses, dissertations, or book chapters; and (3) addressed at least one of the following elements related to mental health recovery‐oriented care planning: concepts, attributes, antecedents, consequences, or methods.

Studies were excluded if they: (1) focused on care planning for non‐mental health conditions; (2) were conference abstracts, study protocols, letters, reports, or other non‐peer‐reviewed publications, with the exception of dissertations and theses, which were eligible for inclusion; (3) addressed care planning focused solely on biomedical treatment; (4) described standalone interventions rather than care planning tools (e.g., the Wellness and Recovery Action Plan); (5) focused exclusively on implementation barriers, facilitators, or organisational factors relating to recovery‐oriented care planning, without examining its concepts, attributes, antecedents, consequences, or methods; or (6) were published in languages other than English and Thai.

All retrieved studies were imported into Covidence (www.covidence.org), where duplicates were removed. N.I. and P.P. independently screened titles and abstracts and subsequently assessed the full texts for eligibility. Any discrepancies or uncertainties were resolved through discussion between the two reviewers (N.I. and P.P.).

### 3.3 Data Extraction

Four reviewers (N.I., P.P., J.K., W.S.) independently conducted data extraction using a predesigned Microsoft Excel data extraction form, with each reviewer responsible for an equal number of studies and cross checks performed to ensure consistency. The extracted information included author, year of publication, country, title, study design, population or sample size, measurement, definition, antecedents, attributes, consequences and empirical referents.

### 3.4 Data Analysis

The extracted data were analysed using Walker and Avant (2005) concept analysis.

#### 3.4.1 Determining the Defining Attributes

Establishing clear conceptual boundaries is considered fundamental in research because it promotes shared understanding among researchers and improves the clarity, consistency and transferability of findings (Stichler 2018). Conceptual clarification aims to refine the meaning of a concept by reducing ambiguity, identifying its core attributes and distinguishing it from overlapping concepts (Bringmann et al. 2022). Therefore, identifying defining attributes, as a key component of concept analysis, was conducted as these attributes represent the essential characteristics that define a concept and differentiate it from related or similar concepts (Gunawan et al. 2023; Walker and Avant 2005). The extracted data related to recovery‐oriented care planning attributes was then analysed and categorised into groups based on their commonality, which were then synthesised to generate the final set of defining attributes of recovery‐oriented care planning.

#### 3.4.2 Identifying the Model Case and Additional Cases

A case study provides an in depth understanding of complex real world phenomena within their natural context by capturing their complexity in a detailed and contextualised manner (Tobita 2025; Yin 2018). After attributes of recovery‐oriented care planning were determined, a model case which is a case example that includes all the defining attributes of a recovery‐oriented care planning was constructed as a real‐life scenario illustrating its key characteristics (Walker and Avant 2005). Additional cases, such as borderline cases (which include some but not all attributes) and contrary cases (which include none of the defining attributes and demonstrate what the concept is not), were also constructed to help explore variations in the concept, clarify its boundaries and distinguish recovery‐oriented care planning from closely related ideas (McKenna 2006; Walker and Avant 2005), for example, other types of care planning.

#### 3.4.3 Identifying Antecedents

Antecedents, defined as circumstances or events occurring prior to recovery‐oriented care planning, were analysed and categorised into groups to situate the concept within its broader social context and identify underlying assumptions (Fitzpatrick and McCarthy 2016; Walker and Avant 2005). In this study, antecedents are understood as factors contributing to the emergence and development of recovery‐oriented care planning.

#### 3.4.4 Identifying Consequences

Consequences, defined as outcomes that occur following the application of recovery‐oriented care planning, were analysed and categorised into groups to examine their effects in practice (Fitzpatrick and McCarthy 2016; Walker and Avant 2005). In this study, consequences refer to outcomes associated with its use, including benefits, barriers, facilitators and other implementation‐related effects.

#### 3.4.5 Defining Empirical Referents

Empirical referents, defined as observable phenomena indicating the presence of a concept, were analysed and categorised into groups to identify how recovery‐oriented care planning is demonstrated in practice (Walker and Avant 2005). In this study, empirical referents are considered as measurable indicators, practical tools and related concepts that reflect their defining attributes within care planning processes.

## 4 Results

### 4.1 Search Results

**FIGURE 1:**
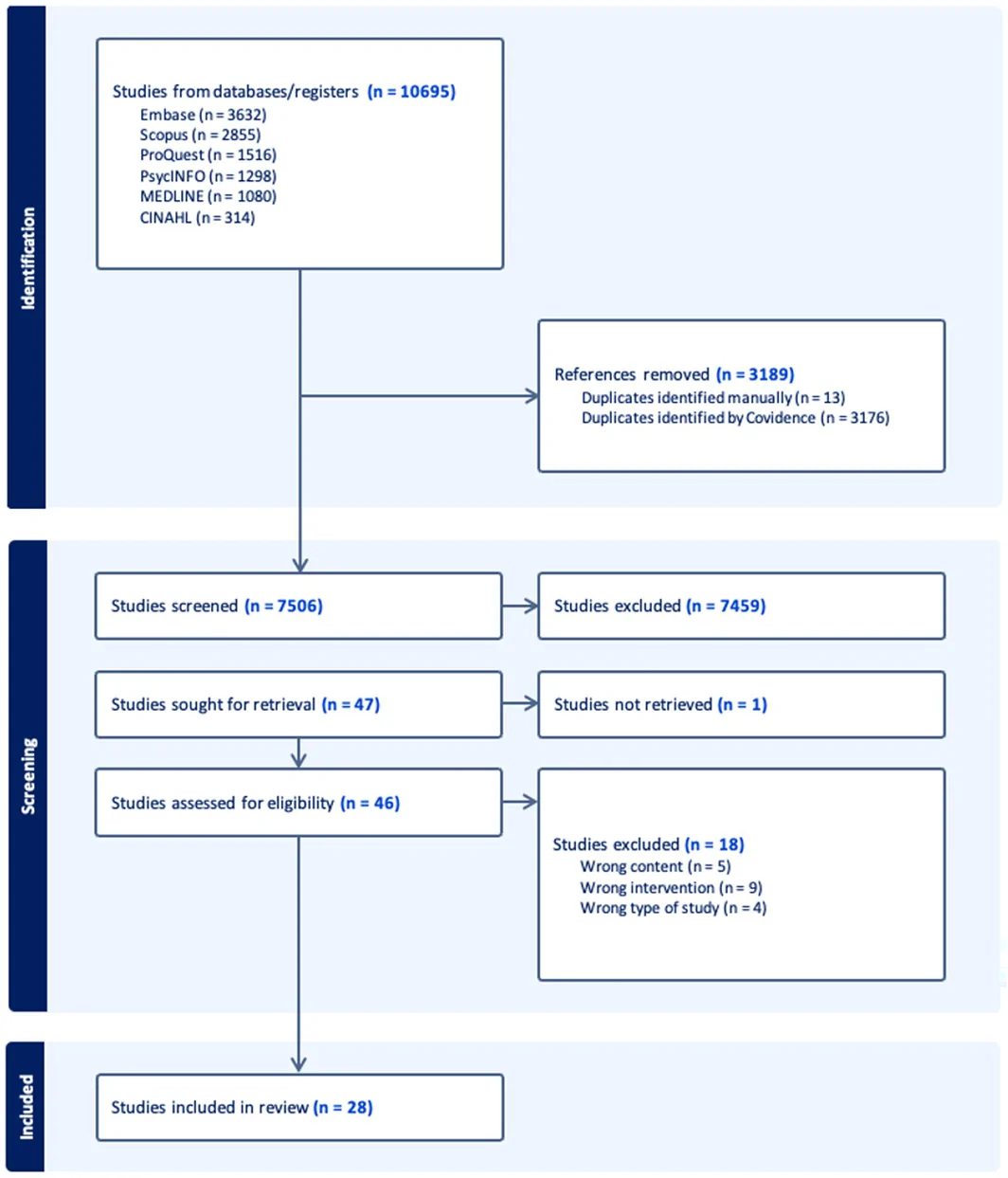
PRISMA flow diagram.

The database search yielded a total of 10 695 articles and included 28 studies in the review. A summary of the systematic search strategy and screening assessment of eligibility criteria is displayed in Figure 1.

### 4.2 Study Characteristics

A total of 28 studies published between 1998 and 2026 were included in this review, originating from the United Kingdom (*n* = 9), Australia (*n* = 8), the United States (*n* = 5), Canada (*n* = 2), Japan (*n* = 1), Ireland (*n* = 1) and Italy (*n* = 1), whilst one book chapter did not report the country of origin. The included literature comprised a wide range of study designs, including qualitative studies, mixed methods studies, retrospective audits, ethnography, quality improvement projects, realist syntheses, systematic and narrative reviews, conceptual and discussion papers, implementation reports and book chapters. Most studies were situated within mental health inpatient, community mental health, or primary care settings and explored recovery‐oriented, person‐centred, or collaborative approaches to care planning. Participants across the studies included service users, mental health professionals, nursing students, carers, peer support workers and organisational leaders, with several studies examining multidisciplinary collaboration and service user involvement in care planning processes. The included studies addressed a range of topics related to recovery‐oriented care planning, including shared decision‐making, collaborative goal setting, implementation barriers and facilitators, workplace culture, therapeutic relationships, recovery‐oriented documentation and the use of recovery‐focused tools and interventions within mental healthcare services. A characteristic table is presented in Data S1.

### 4.3 Definition of Mental Health Recovery‐Oriented Care Planning

The reviewed studies consistently described recovery‐oriented care planning as a collaborative, person‐centred and strengths‐based process rather than a single clinical activity or standardised documentation procedure. It was viewed as an ongoing and individualised process that supports individuals in identifying and achieving meaningful recovery goals through the development and implementation of action plans (Berger 2006; Dadich et al. 2013; John 2022; Lodge et al. 2017; Mancini 2016; Miller et al. 2017; O'Donohue et al. 2023; Shue et al. 2023; Wyder et al. 2018). Recovery goals and action plans were typically self‐directed, personally meaningful and developed collaboratively with service users to promote dialogue and shared decision‐making, with a focus on individuals' goals and aspirations rather than problems alone (Branjerdporn et al. 2023; Dadich et al. 2013; Reid et al. 2018).

Recovery‐oriented care planning also extended beyond the relationship between service users and healthcare professionals by involving families and other support networks through partnership working and collaborative decision making where appropriate (Branjerdporn et al. 2023; Lodge et al. 2017; McHugh and Byrne 2012; Shue et al. 2023). Unlike traditional clinician‐driven treatment planning, where symptom management is prioritised, recovery‐oriented care planning emphasised individuals' strengths, values, aspirations and social recovery (Dadich et al. 2013; Rinaldi and Watkeys 2014; Tondora et al. 2012). Healthcare professionals were therefore positioned less as experts directing care and more as collaborators, facilitators, or coaches supporting individuals in working towards personally meaningful goals, recognising service users as experts in their own lives and experiences (Borkman 1998; John 2022). Furthermore, recovery‐oriented care planning was viewed as a flexible and dynamic process that should avoid becoming routine, professionally dominated, or bureaucratic (Borkman 1998; O'Donohue et al. 2023).

### 4.4 Antecedents of Recovery‐Oriented Care Planning

From the reviewed studies, we identified several antecedents that contributed to the emergence and development of recovery‐oriented care planning within mental healthcare services. These antecedents reflected broader philosophical, policy, organisational and practice shifts towards recovery‐oriented, person‐centred and collaborative approaches to care.

#### 4.4.1 Shift Towards Recovery‐Oriented and Person‐Centred Mental Healthcare

The literature consistently identified recovery‐oriented care planning as emerging from broader philosophical, policy and service reforms within mental healthcare. This shift reflected movement away from paternalistic, illness‐focused and clinician‐driven models towards recovery‐oriented, person‐centred and collaborative approaches (Berger 2006; Ebihara et al. 2026; Hipfner et al. 2017; John 2022; Marston and Weinstein 2013; Tondora et al. 2012). National and international policies and standards increasingly promoted collaborative, personalised and recovery‐focused care planning, including initiatives from the American Psychiatric Association (Branjerdporn et al. 2023), the Mental Health Commission of Canada (Hipfner et al. 2017) and the Australian mental health standards (Palmer et al. 2014), alongside broader deinstitutionalisation, psychiatric rehabilitation, community mental healthcare and person‐centred care movements (Dadich et al. 2013; Jones et al. 2018). Consequently, recovery‐oriented care planning emerged to promote autonomy, empowerment, self‐determination and meaningful participation in care rather than focusing primarily on symptom reduction.

#### 4.4.2 Critique of Traditional Care Planning and the Need for Holistic Recovery Support

A major antecedent across the literature was dissatisfaction with traditional care planning practices, which were frequently described as professionally controlled, risk‐focused, bureaucratic and centred on diagnosis, medication and organisational priorities rather than individuals' aspirations and meaningful life goals (Borkman 1998; John 2022; Reid et al. 2018; Rinaldi and Watkeys 2014). Service users and carers commonly reported feeling excluded, marginalised, passive and distanced from decision‐making processes (Borkman 1998; Dadich et al. 2013; John 2022; Lambert 2019; Wyder et al. 2018). Traditional approaches were also criticised for poor multidisciplinary collaboration, inadequate communication and limited attention to broader psychosocial and quality‐of‐life domains important to recovery, such as housing, employment, relationships, independence and community participation (Jones et al. 2018; Mancini 2016; Rylance and Graham 2014). Recovery‐oriented care planning therefore emerged in response to the need for more holistic, strengths‐based and collaborative approaches involving service users, families, carers, multidisciplinary teams and community supports.

### 4.5 Defining Attributes of Recovery‐Oriented Care Planning

There were several defining attributes, drawing from included literature, that characterise recovery‐oriented care planning in practice. These attributes reflected the collaborative, person‐centred, strengths‐based and recovery‐focused nature of care planning processes and relationships between service users, healthcare professionals, families and other supports. Table 1 presents each defining attribute of recovery‐oriented care planning.

**TABLE 1:** Defining attributes of recovery‐oriented care planning.

Main attributes	Key defining attributes	Examples from included studies
1. Collaboration, shared decision‐making and service user involvement	Collaborative care planning; shared decision‐making; partnership working; multidisciplinary involvement; inclusion of families, carers, peer support workers and natural supports; service users as active participants and experts by experience	− Putting the person in the driving seat in relation to the interaction (Berger 2006) − Decision making is shared between providers, people in recovery and their families (Miller et al. 2017) − The interventions/strategies to achieve these goals were then formulated by the nurse asking the question ‘How can we achieve these together?’ (Reid et al. 2018) − Recovery planning is framed as a consumer‐clinician collaborative process (Wyder et al. 2018)
2. Person‐centred, strengths‐based and individualised care	Focus on strengths, hopes, aspirations, interests, values and personal goals; holistic understanding of the person; avoiding deficit‐focused and diagnosis‐led approaches; culturally sensitive and personalised care	− Looking at people in their entirety and does not endorse defining them by their diagnosis (Hipfner et al. 2017) − The plan should be built around the person's own life goals (Tondora et al. 2012) − It is these strengths, hopes and aspirations that should underpin and inform care planning (Marston and Weinstein 2013) − A strengths‐based conceptualisation of service users that recognises their potential (McHugh and Byrne 2012)
3. Recovery‐focused goal setting and action planning	Personally meaningful recovery goals; action‐oriented planning; measurable objectives; timelines and responsibilities; focus on life goals beyond symptom reduction	− Recovery goals are a person's own goals—Recovery goals are identified by the person (Rinaldi and Watkeys 2014) − Measurable short‐term objectives linked to long‐term life goals (Tondora et al. 2012) − The weekly recovery plan is a concrete list of objectives, actions and steps (Borkman 1998) − Focuses on identifying goals, split into specific actions (Pithara et al. 2020)
4. Therapeutic, hopeful and empowering relationships	Compassionate and respectful therapeutic relationships; hope promotion; empowerment; self‐management; professionals acting as facilitators, collaborators, or coaches rather than directors of care	− Inspiring hope through designing a realistic future together (Berger 2006) − The nurse's role as an active one by describing care planning as a ‘helping’ conversation between equals (Lambert 2019) − Professionals through coproduction support people's confidence and skills enabling them to self‐manage (Rinaldi and Watkeys 2014) − Repositioning service users as empowered participants in their own recoveries (Mancini 2016)
5. Dynamic, flexible and living process	Ongoing and evolving process; flexibility; continuous review and adaptation; therapeutic process rather than bureaucratic documentation	− Being a ‘living’ recovery care plan for each service user (McHugh and Byrne 2012) − It needs to be a living document added to each day (Marston and Weinstein 2013) − The planning process itself is therapeutic (Tondora et al. 2012) − A dynamic and collaborative process underpinned by self‐management, shared decision making and coproduction (Rinaldi and Watkeys 2014)

#### 4.5.1 Collaboration, Shared Decision‐Making and Service User Involvement

A central defining attribute across our included literature was collaboration between service users, healthcare professionals, families, carers and other supports throughout the care planning process (Jones et al. 2018; Miller et al. 2017; Rio et al. 2021; Tondora et al. 2012; Wyder et al. 2018). Recovery‐oriented care planning was consistently described as a collaborative, shared and person‐led process that positioned service users as active participants rather than passive recipients of care (Lambert 2019; McHugh and Byrne 2012; Reid et al. 2018). Shared decision‐making, mutual dialogue, negotiation and partnership working were repeatedly emphasised, alongside recognition of service users as experts in their own lives and experiences (Berger 2006; Mancini 2016). Several studies also highlighted the importance of involving multidisciplinary teams, peer support workers, families and natural supports in planning processes (John 2022; Kisely et al. 2017; O'Donohue et al. 2023).

#### 4.5.2 Person‐Centred, Strengths‐Based and Individualised Care

The reviewed studies consistently highlighted person‐centredness, strengths‐based practice and individualisation as core attributes of recovery‐oriented care planning (Hipfner et al. 2017; Lambert 2019; McHugh and Byrne 2012; Miller et al. 2017; Tondora et al. 2012). Care plans were expected to focus on individuals' strengths, hopes, aspirations, interests, values and personal goals rather than deficits, diagnoses, symptoms, or organisational priorities (Marston and Weinstein 2013; Murphy and Cutts 2009; Rinaldi and Watkeys 2014). Recovery‐oriented care planning also emphasised understanding individuals holistically, including their cultural context, relationships, spirituality and broader life domains such as employment, housing, education and community participation (Branjerdporn et al. 2023; Marston and Weinstein 2013; Miller et al. 2017; Wyder et al. 2018).

#### 4.5.3 Recovery‐Focused Goal Setting and Action Planning

Another defining attribute was the emphasis on personally meaningful recovery goals and practical action planning (Borkman 1998; Pithara et al. 2020; Rinaldi and Watkeys 2014; Tondora et al. 2012). Recovery‐oriented care planning involved identifying long‐term recovery goals, breaking these into achievable short‐term objectives, and collaboratively developing action plans, strategies, timelines and responsibilities (Berger 2006; McHugh and Byrne 2012). Goals extended beyond symptom reduction to include meaningful life roles, independence, wellness, employment, education, social participation and community living (Miller et al. 2017; O'Donohue et al. 2023; Wyder et al. 2018).

#### 4.5.4 Therapeutic, Hopeful and Empowering Relationships

The reviewed literature further identified therapeutic relationships characterised by compassion, respect, listening, encouragement and hope as essential attributes of recovery‐oriented care planning (Berger 2006; Lambert 2019; Reid et al. 2018). Healthcare professionals were generally described taking on roles as collaborators, facilitators, coaches, or supporters who enable service users' recovery rather than directing or controlling care (Mancini 2016; Rinaldi and Watkeys 2014). Recovery‐oriented care planning also aimed to promote autonomy, empowerment, self‐management, confidence, responsibility and hope (Ebihara et al. 2026; Palmer et al. 2014).

#### 4.5.5 Dynamic, Flexible and Living Process

Recovery‐oriented care planning was also characterised as an ongoing, dynamic and flexible process rather than a static or bureaucratic document (McHugh and Byrne 2012; Rinaldi and Watkeys 2014; Simpson et al. 2016). Care plans should evolve with individuals' changing circumstances, recovery journeys and preferences (Marston and Weinstein 2013; Rinaldi and Watkeys 2014). The planning process itself was described as therapeutic, relational and continuously reviewed through collaborative engagement (Rio et al. 2021; Tondora et al. 2012).

### 4.6 The Model Case and Additional Cases of Recovery‐Oriented Care Planning

To further illustrate the defining attributes of recovery‐oriented care planning identified across the included literature, a model case and additional cases were constructed. These cases demonstrate how the concept may appear in practice, including examples reflecting all defining attributes (model case), some but not all attributes (borderline case) and an example that does not reflect the defining attributes of recovery‐oriented care planning (contrary case).

#### 4.6.1 A Model Case

Mr. Liam is a 32‐year‐old man living with schizophrenia who attends a community mental health service. During a care planning meeting, Mr. Liam, his nurse, his mother, and a peer support worker collaboratively discuss his recovery goals. Mr. Liam explains that he wants to return to part‐time work, reconnect with friends and improve his confidence in managing his medication independently. Together, they develop a recovery‐oriented care plan using Mr. Liam's own words, identifying his strengths, interests in cooking and supportive family relationships. The plan includes achievable short‐term goals, practical action steps and regular review meetings. The nurse acts as a facilitator and encourages Mr. Liam to make decisions about his care whilst providing hope and support. The care plan is reviewed regularly and adapted according to Mr. Liam's progress and changing needs.

This case demonstrates all defining attributes of recovery‐oriented care planning, including collaboration, shared decision‐making, person‐centred and strengths‐based care, recovery‐focused goal setting, empowering therapeutic relationships and an ongoing dynamic process.

#### 4.6.2 A Borderline Case

Ms. Yvonne is a 40‐year‐old woman with bipolar disorder receiving outpatient care. During an appointment, her psychiatrist asks about her goals and records that she would like to improve her daily routine and reduce stress. The care plan includes medication management and a referral for counselling. Although Ms. Yvonne is involved in discussing some goals, most decisions are made by the psychiatrist, and the plan mainly focuses on symptom control and treatment adherence. The care plan is documented professionally and reviewed infrequently, with limited involvement from family or other supports.

This case includes some attributes of recovery‐oriented care planning, such as limited collaboration and personal goal identification, but lacks broader strengths‐based, holistic, empowering and fully collaborative elements.

#### 4.6.3 A Contrary Case

Mr. Anan is admitted to an inpatient psychiatric ward following a mental health crisis. The multidisciplinary team develops a care plan without discussing it with him. The plan focuses primarily on risk management, medication compliance, behavioural monitoring and discharge procedures. Mr. Anan's personal goals, strengths, preferences and social circumstances are not explored. He is informed of the care plan after it has already been completed and has no opportunity to contribute to decisions about his treatment. The care plan remains unchanged throughout admission unless his symptoms worsen.

This case demonstrates none of the defining attributes of recovery‐oriented care planning. The process is clinician‐driven, deficit‐focused, non‐collaborative and centred on risk and symptom management rather than recovery, empowerment and partnership working.

### 4.7 Consequences of Recovery‐Oriented Care Planning

The consequences associated with recovery‐oriented care planning, identified across included studies, reflected the impact of recovery‐oriented care planning on service users, therapeutic relationships, collaborative working and the delivery of more personalised and recovery‐focused mental healthcare.

#### 4.7.1 Enhanced Service User Empowerment and Recovery

The studies reported that recovery‐oriented care planning promoted greater service user involvement, autonomy, empowerment and ownership of recovery processes. Service users described feeling more listened to, respected, acknowledged and actively involved in decisions about their care (Berger 2006; Wyder et al. 2018). Collaborative care planning supported individuals to identify meaningful aspirations, formulate achievable goals and develop strategies for recovery and self‐management (Dadich et al. 2013; Reid et al. 2018). Recovery‐oriented care planning was also associated with increased hope, confidence, self‐management, responsibility and sense of purpose by linking treatment to meaningful life goals (Rinaldi and Watkeys 2014; Tondora et al. 2012). Several studies further reported improvements in empowerment, quality of life, medication adherence, social participation, community integration and psychological wellbeing (Lambert 2019; Miller et al. 2017; Palmer et al. 2014).

#### 4.7.2 Improved Therapeutic Relationships and Collaborative Care

Recovery‐oriented care planning strengthened therapeutic relationships and collaboration between service users, families and healthcare professionals. The studies highlighted improvements in communication, dialogue, mutual understanding and shared decision‐making through collaborative planning processes (Dadich et al. 2013; Hipfner et al. 2017; Murphy and Cutts 2009). The roles of healthcare professionals were moved from expert‐driven towards more collaborative and supportive partnerships with service users (Ebihara et al. 2026). Recovery‐oriented care planning also improved continuity of care, multidisciplinary collaboration, advocacy and coordination between inpatient and community services (Jones et al. 2018; Marston and Weinstein 2013).

#### 4.7.3 More Individualised and Holistic Care

The literature indicated that recovery‐oriented care planning supported more personalised, strengths‐based and holistic care beyond symptom management alone. Recovery‐oriented care plans encouraged greater focus on individuals' strengths, recovery goals, housing, employment, education, relationships, meaningful activities and community participation (Miller et al. 2017; Wyder et al. 2018). Several studies also highlighted increased use of first‐person, strengths‐based and individualised language, contributing to more meaningful and person‐centred care planning (Hipfner et al. 2017; Simpson et al. 2016; Wyder et al. 2018).

**FIGURE 2:**
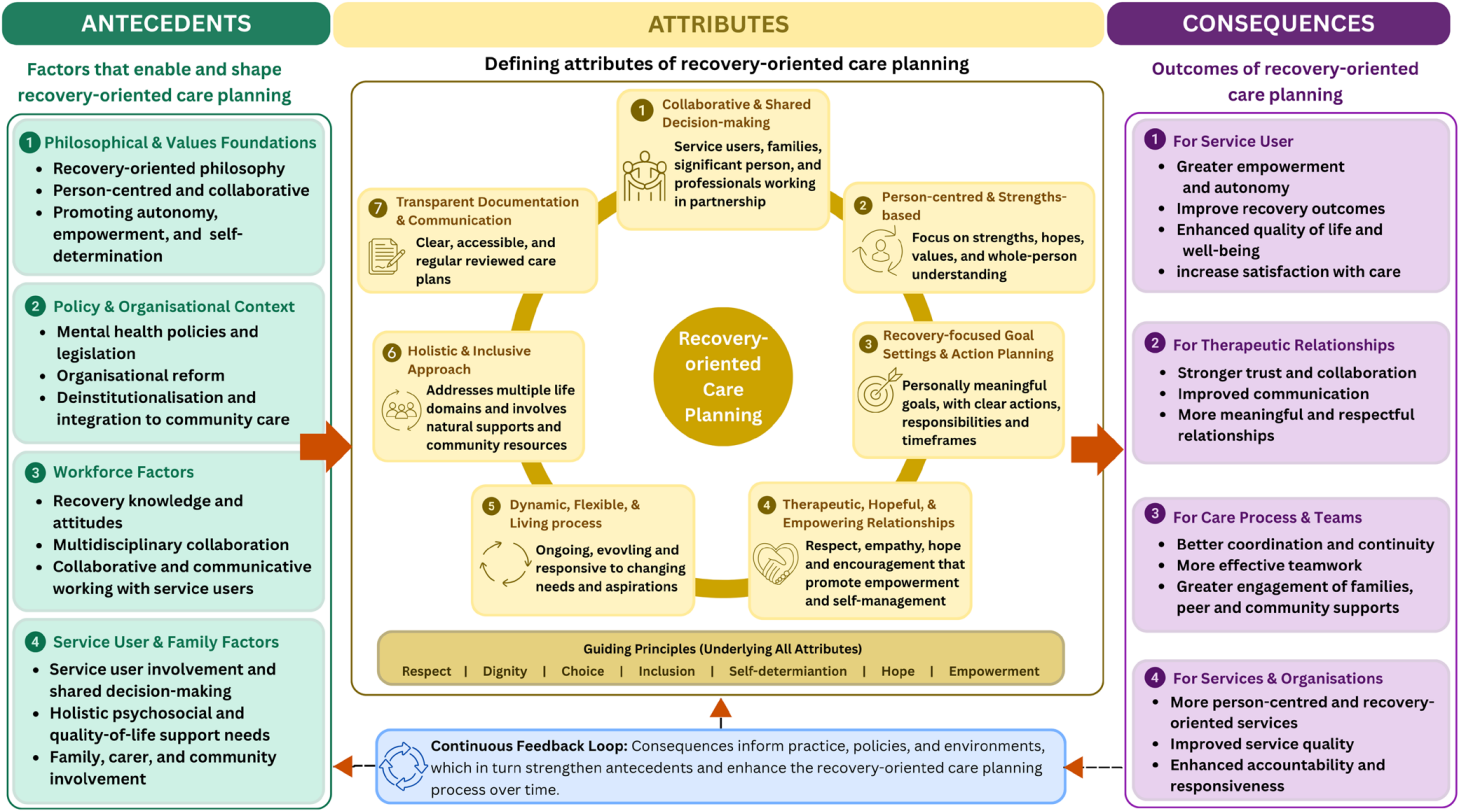
A conceptual model of recovery‐oriented care planning (Antecedents‐attributes‐consequences).

A proposed conceptual model developed from the findings on the antecedents, attributes and consequences of recovery‐oriented care planning is presented in Figure 2. The model illustrates how these components interrelate to define the concept of recovery‐oriented care planning.

### 4.8 Empirical Referents of Recovery‐Oriented Care Planning

The literature identified several observable and measurable indicators demonstrating the presence of recovery‐oriented care planning in practice. These empirical referents commonly included the use of structured recovery‐oriented care planning tools, documentation approaches, collaborative goal‐setting processes and recovery‐focused outcome measures.

#### 4.8.1 Recovery‐Oriented Care Planning Tools and Documentation

Across included literature, there are several structured tools and documentation systems identified as empirical referents of recovery‐oriented care planning in practice. These included master and weekly recovery plans (Borkman 1998), Joint Crisis Plans (Ebihara et al. 2026), the Tidal Model concept map (Hipfner et al. 2017), the Care Programme Approach (CPA) (John 2022), Wellness Recovery Action Plans (WRAPs) (Palmer et al. 2014; Simpson et al. 2016), Recovery Star tools (Lambert 2019) and the Mental Health Recovery Star (Jones et al. 2018; Martinelli et al. 2023). These tools were used to support recovery goal setting, collaborative planning, self‐management, crisis planning and continuity of care.

#### 4.8.2 Collaborative and Recovery‐Focused Care Planning Process

Several empirical referents reflected the collaborative and recovery‐oriented nature of the care planning process itself. These included collaborative goal setting between service users and healthcare professionals, documentation of personal recovery goals, shared decision‐making, use of first‐person and strengths‐based language and greater involvement of service users in understanding, reviewing and shaping their own care plans (Lambert 2019; Rinaldi and Watkeys 2014). Recovery Colleges and educational initiatives were also described as supporting service users' participation, self‐management and understanding of recovery‐oriented care planning processes (Rinaldi and Watkeys 2014).

#### 4.8.3 Recovery‐Oriented Outcome and Fidelity Measures

The literature additionally identified measurable indicators used to assess recovery‐oriented care planning practices and fidelity. These included the Person‐centred Care Questionnaire, Recovery Knowledge Inventory and Recovery Self‐Assessment Scale, which were used to evaluate providers' recovery‐oriented practice, organisational recovery orientation and adherence to person‐centred care planning principles (Miller et al. 2017). Online personal health records and integrated care documentation systems were also identified as practical indicators of collaborative and recovery‐focused care planning within routine mental healthcare practice (Rinaldi and Watkeys 2014).

## 5 Discussion

This concept analysis clarified recovery‐oriented care planning as an individualised process developed with service users, involving people connected to the individual throughout the care planning process, and remaining flexible and responsive to their changing needs, with healthcare professionals acting as facilitators within a collaborative, person‐centred and strengths‐based approach that extends beyond traditional symptom‐oriented treatment planning. However, we identified substantial conceptual overlap between recovery‐oriented care planning, person‐centred care planning and collaborative care planning, reflecting broader conceptual ambiguity within the recovery‐oriented mental healthcare literature. Although our concept analysis supported the identification of defining attributes of recovery‐oriented care planning and facilitated differentiation between recovery‐oriented care planning and related approaches, the shared philosophical foundations and common emphasis on partnership, individualisation, and holistic care across these concepts made them difficult to distinguish as each approach to care planning may designate a specific focus. For example, person‐centred care planning not only focuses on supporting each individual's unique life goals, aspirations, capacities, strengths and interests, but also aims to promote recovery and meaningful living within the community of the person's choice (Davidson and Tondora 2022). Similarly, collaborative care planning emerged from efforts to move away from fragmented, paternalistic and disease‐oriented models towards more relationship‐based, collaborative and holistic approaches to care and is therefore closely aligned with person‐centred care principles (Jobe et al. 2020). As recovery itself is widely understood as a deeply personal, meaningful and individualised journey, recovery‐oriented practice inherently shares many principles with person‐centred and collaborative approaches (Swords and Houston 2024). Nevertheless, recovery‐oriented care planning may be distinguished by its more explicit orientation towards personal recovery and its emphasis on supporting Connectedness, Hope, Identity, Meaning and Empowerment, as conceptualised within the CHIME framework (Leamy et al. 2011). In this sense, recovery‐oriented care planning appears to integrate elements of person‐centred and collaborative care planning whilst placing stronger emphasis on supporting meaningful recovery beyond symptom management alone, as the perceived outcome of clinical recovery.

The findings highlighted ongoing tensions between recovery‐oriented values and the realities of contemporary mental healthcare systems. Although recovery‐oriented care planning is frequently promoted within policy frameworks internationally (Inta, Grealish, et al. 2026; Inta, Leamy, et al. 2026), we found that care planning processes remained heavily shaped by biomedical priorities, organisational bureaucracy, audit requirements and risk‐focused cultures. This reflects wider implementation literature suggesting that recovery‐oriented practice often becomes incorporated into existing systems without fundamentally changing underlying professional assumptions or power structures (Crowe 2022; Slade et al. 2014). In this sense, recovery‐oriented care planning may risk becoming another procedural requirement unless supported by broader cultural and organisational transformation. Our proposed conceptual model of the recovery‐oriented care planning process identifies key components for consideration and highlights that the format of documentation is less important than ensuring that the content reflects what is personally meaningful and prioritised by the individual. The findings therefore support arguments that recovery‐oriented practice is not simply the introduction of new documentation tools or terminology, but a deeper shift in professional identity, relationships and understandings of expertise within mental healthcare.

The defining attributes identified across the reviewed studies further reinforced these tensions. Although collaboration and shared decision‐making were consistently described as central characteristics of recovery‐oriented care planning, the findings simultaneously revealed that genuine collaboration remains difficult to achieve in practice. Service users and carers frequently reported feeling excluded, marginalised, or positioned as passive participants despite policies promoting involvement. This contrast between policy rhetoric and lived experience has also been highlighted within wider recovery and person‐centred care literature, where shared decision‐making is often advocated theoretically but constrained in practice by time pressures, organisational hierarchies, professional dominance and concerns about risk and liability (Ahmed et al. 2021; Waddell et al. 2021). The findings therefore suggest that collaborative care planning should not be understood as simply asking service users about goals or preferences, but as involving meaningful negotiation, shared power and recognition of experiential knowledge.

The consequences identified across the reviewed studies further demonstrated the potential value of recovery‐oriented care planning. Recovery‐oriented care planning was associated with greater empowerment, autonomy, hope, self‐management, therapeutic alliance and service user participation. These findings are consistent with wider evidence suggesting that collaborative and recovery‐focused approaches may improve service user engagement, satisfaction and psychosocial outcomes (Ezaydi et al. 2023; Stewart et al. 2022). Importantly, our study also highlighted relational and organisational consequences, including improved communication, multidisciplinary collaboration, continuity of care and more individualised support. However, we found that these positive outcomes are not automatically achieved through the introduction of recovery‐oriented care planning tools alone. Rather, the effectiveness of recovery‐oriented care planning appeared highly dependent on whether recovery principles are genuinely embedded within professional practice and organisational culture.

### 5.1 Strengths and Limitations

This concept analysis has several strengths. First, the integration of concept analysis with a scoping review approach enabled a systematic and transparent synthesis of the literature relating to recovery‐oriented care planning. This approach enhanced conceptual clarification by drawing on diverse sources across policy, theoretical and empirical literature. Second, the use of the concept analysis framework of Walker and Avant (2005) provided a structured process for identifying antecedents, defining attributes, consequences, model cases and empirical referents, which helped distinguish recovery‐oriented care planning from related concepts such as person‐centred care planning and collaborative care planning. Third, the inclusion of model, borderline and contrary cases provided practical illustrations of how recovery‐oriented care planning may manifest in real‐world practice, thereby improving conceptual understanding and applicability within mental healthcare settings.

Several limitations should also be acknowledged. Given the nature of a scoping review, no formal quality appraisal of the included studies was conducted, which may raise concerns regarding potential risk of bias. However, all studies were systematically extracted and analysed in accordance with the principles of concept analysis. The reviewed literature originated predominantly from high‐income Western countries, particularly the United Kingdom, Australia, Canada and the United States, which may limit the transferability of findings to non‐Western or low‐ and middle‐income settings where cultural understandings of recovery, autonomy, family involvement and professional roles may differ. In addition, substantial conceptual overlap was identified between recovery‐oriented care planning, person‐centred care planning, collaborative care planning and strengths‐based approaches, reflecting ongoing conceptual ambiguity within the literature. The findings were also limited by variability in terminology, theoretical positioning and operationalisation across included studies, making direct comparison challenging. Furthermore, as concept analysis is interpretive in nature, identification and synthesis of defining attributes may be influenced by researchers' interpretation despite efforts to maintain systematic and transparent analysis procedures.

This scoping review included both primary studies and review articles to provide a comprehensive overview of the literature. Although review articles were synthesised at the review level rather than by re‐extracting data from their included primary studies to minimise duplication, some overlap between evidence sources may still have occurred. In addition, findings derived from review articles represent secondary interpretations of primary evidence, meaning that this review is further removed from the original data and may therefore inherit biases or inaccuracies present in the included reviews. However, conclusions were drawn cautiously, with these limitations considered in the interpretation of the findings, in accordance with the principles of concept analysis. These considerations should be taken into account when interpreting our findings.

### 5.2 Implications for Future Research

Several implications arise from this study. First, recovery‐oriented care planning appears to require more than procedural or documentation changes; it involves relational, cultural and organisational transformation within mental healthcare systems. Second, implementing recovery‐oriented care planning may require healthcare professionals to critically reflect on traditional professional roles, authority and assumptions regarding expertise and risk. Third, the findings highlight the importance of ensuring that recovery‐oriented care planning remains flexible, meaningful and person‐led rather than becoming absorbed into bureaucratic documentation processes. The value of a care plan lies not in the format used, but in whether its content reflects the individual's priorities, values, strengths and recovery goals. Mental health services should therefore focus on the quality of collaborative conversations and therapeutic relationships that underpin care planning, rather than solely on documentation requirements and compliance. Finally, given the dominance of Western literature within this review, further research is needed to explore how recovery‐oriented care planning may be understood, adapted and operationalised within diverse cultural and healthcare contexts, particularly in low‐ and middle‐income countries where understandings of recovery, autonomy, family involvement and professional relationships may differ substantially from Western recovery models.

### 5.3 Relevance for Clinical Practice

This concept analysis provides greater clarity regarding the meaning and key components of recovery‐oriented care planning. The findings may support healthcare professionals in translating recovery principles into everyday practice by promoting shared decision‐making, meaningful recovery goal setting, strengths‐based approaches and active service user involvement. The proposed conceptual model may also guide the development, implementation and evaluation of recovery‐oriented care planning within mental healthcare services.

## 6 Conclusion

This concept analysis clarified recovery‐oriented care planning as an individualised process developed with service users, involving people connected to the individual throughout the care planning process, and remaining flexible and responsive to their changing needs, with healthcare professionals acting as facilitators within a collaborative, person‐centred and strengths‐based approach that extends beyond traditional symptom‐oriented treatment planning. The findings highlight that recovery‐oriented care planning is characterised by shared decision‐making, meaningful recovery goal setting, empowering therapeutic relationships and holistic support that promotes autonomy, hope and participation in recovery. This study also demonstrated that recovery‐oriented care planning emerged in response to limitations of traditional clinician‐led and risk‐focused approaches and can be operationalised through various recovery‐oriented tools, practices and measurable indicators. Overall, this concept analysis provides greater conceptual clarity of recovery‐oriented care planning, which could be used to support future research, education, policy and its implementation within mental healthcare services.

## Author Contributions

**Natthapon Inta:** conceptualisation, investigation, methodology, formal analysis, validation, data curation, visualisation, project administration, writing – original draft, writing – review and editing. **Prapan Phetlerthirunkul:** investigation, validation, data curation, writing – review and editing. **Jirada Kaewchuchuen:** investigation, validation, writing – review and editing. **Chonmanan Khanthavudh:** validation, writing – review and editing. **Wilailak Sakharang:** conceptualization, investigation, validation, data curation, writing – review and editing.

## Funding

The authors have nothing to report.

## Conflicts of Interest

The authors declare no conflicts of interest.

## Supporting information


**Data S1:** Characteristics of included studies according to author name, ordered alphabetically (*N* = 28).

## Data Availability

The data that supports the findings of this study are available in the Supporting Information of this article.
