# Supplementary material for: Recovery‐Oriented Care Planning in Mental Healthcare Services: A Combined Concept Analysis and Scoping Review
Source: Int J Ment Health Nurs. 2026 Aug 19;35(4):e70337. doi: 10.1111/inm.70337 (PMC13490880; doi:10.1111/inm.70337)
Supplement: Supplementary file 1 [file INM-35-0-s001.docx]

**Supplementary File S1: Characteristics of included studies according to author name, ordered alphabetically (*N*=28).**

| **Author(s), year Country** | **Title** | **Aim/objectives/research questions** | **Study design / Research type** | **Population  (sample size)** | **Measurement of recovery outcome** | **Main finding** |
| --- | --- | --- | --- | --- | --- | --- |
| Berger (2006)  Canada | Incorporation of the tidal model into the interdisciplinary plan of care - a program quality improvement project | To evaluate the impact of the new Interdisciplinary Care Plan (IPC) | A quality improvement project  (Qualitative + Quantitative) | Patients and staff in acute mental health setting in Canada | **Clients:** - The Perceptions of Care questionnaire (Eisen et al. 2002) measuring clients' satisfaction | Feedback demonstrated that the IPC had a positive impact on both client and caregiver satisfaction, provided a better understanding of the client’s situation, and improved the team’s ability to document a care plan that reflected the client’s specific goals and the goals of the interdisciplinary team. |
| Borkman (1998)  USA | Is Recovery Planning Any Different from Treatment Planning? | To compare the “recovery” planning process of the social model programs with the “treatment” planning process in a comparison medical model program | Secondary data analysis  (data from the process evaluation) | Patients with substance misuse, and residents in recovery home (support accommodation) | N/R | Functions of treatment planning in the MMP and recovery planning in the SMPs seem to be similar: providing direction for staff and clients, tailoring the program to fit the individual’s needs, providing staff opportunities to know patients/residents, and satisfying the requirements of third-party funders. In both cases, the planning process and documentation constitute a set of objectives and the means for achieving each objective that are tailored for an individual patient/resident during his/her program stay. |
| Branjerdporn et al. (2023)  Australia | Understanding care plans in a psychiatric mother-baby unit | To explore the content of care planning goals, compare against the World Health Organisation’s (WHO) International Classification of Functioning, Disability, and Health (ICF), and devise a care plan framework to support development of admission goals | Retrospective audit | The analysis of 63 care plans across admission, mid-admission and discharge in mother and baby units. | N/R | When compared to the WHO ICF codes, care plans were most commonly coded against d570 (looking after one’s health) and d7600 (parent-child relationships). Care plans covered six main themes: mental health recovery, physical health, connecting with baby, caring for baby, relationships, and community supports. |
| Dadich et al. (2013)  Australia | How can non-clinical case management complement clinical support for people with chronic mental illness residing in the community? | To explore how clinical and non-clinical case managers worked together in consumer care planning and examines the perceived influence of support | Qualitative interviews and document (care plan) analysis | People with chronic mental illness who reside in the community | N/R | This research suggests that collaboration requires consumer-driven planning; inclusive, active partnership participation; planning as a process, including reflective practice; and a shared understanding and commitment to psychosocial support and respective roles within the recovery process. The findings have the potential to inform future complementary case management practices for the benefit of consumer recovery. |
| Ebihara et al. (2026)  Japan | Exploring the Process of Professional Role Redefinition Towards Recovery-Oriented Care Through Joint Crisis Plans in Japan: A Qualitative Study Using the Modified Grounded Theory Approach | To elucidate the experiences of professionals utilising Joint Crisis Plans (JCPs) across diverse facility types  To develop a theoretical understanding of the process by which they redefine professionals’ role from ‘manager’ to ‘recovery companion’ | A qualitative study: the Modified Grounded Theory Approach | 13 professionals (7 nurses, 6 mental health and welfare workers) across nine facilities (psychiatric hospitals, 24-h residential facilities, outpatient facilities) in the Kanto region of Japan | N/R | Nine categories and 23 subcategories were extracted. A three-stage support transformation process emerged:  (1) Initial Stage and Motivation, in which professionals confronted the limitations of managerial practice;  (2) Role Redefinition and Practice through Collaboration, involving joint crisis management, strength-based support, and network building;  (3) Stage of Integration of Perspectives and Implementation of Recovery-Oriented Practice, in which professionals witnessed individual recovery and integrated new support values into their practice. |
| Hipfner et al. (2017)  Canada | Teaching recovery principles with concept map care planning | To describe the integration of the concept map within a tidal/recovery framework | Report | Nursing students (first year) | N/R | Conceptual model (Tidal Model) designed by psychiatric nursing educators to help psychiatric students integrate the recovery principles with the service user’s care plan. |
| John (2022)  UK | Service User Involvement in Recovery-oriented Care Planning: A Realist Synthesis | To practice work best, in what circumstances, and to what extent, to embed an active role for service users’ involvement in recovery-oriented care planning during acute inpatient care | Thesis: A realist synthesis | Mental health staff who had experience in delivering acute inpatient care and service users. | N/A | Five programme theories relating to the acute care pathway were identified following the realist synthesis:  1) ‘Provider-controlled care transition’ (admission to acute inpatient units), referring to the limitations to service users’ active involvement at this stage; 2) ‘Care plan as a recovery tool?’ – addressing the infrastructural and organisational limitations to active SUI in recovery-oriented care-plan formulation; 3) ‘Ward rounds as a non- inclusive arena for shared decision making’, highlighting their unfulfilled potential for shared decision making about treatment;  4) ‘Peer support worker intervention as a key factor in service users’ recovery’, concerning their positive impact; and  5) ‘Provider-controlled care transition from acute inpatient units’ (discharge practice), highlighting limitations of current practice in preparing service users for transition into the community. |
| Jones et al. (2018)  UK | Traditions of research in community mental health care planning and care coordination: A systematic meta-narrative review of the literature | To explore which interventions have proved more or less effective in promoting personalised, recovery-oriented care planning and coordination for community mental health service users. | A systematic review | N/A | In the UK literatures: (1) Carers’ & Users’ Expectation of Services—User version (CUES-U)— 17 item service user outcomes scale.  (2) The Mental Health Recovery Star (MHRS)—outcome measure rated collaboratively by staff and service users assessing 10 life domains. * Assesses social function more than recovery. Concluded that the MHRS cannot be recommended as a routine clinical outcome tool.  In the international literatures: (3) PNCQ (Perceived need for care questionnaire), a self-rated needs assessment tool | The review found no seminal papers in terms of high citation rates, or papers that were consistently cited over time. The traditions of research in this topic area have formed reactively in response to frequent and often unpredictable policy changes, rather than proactively as a result of intrinsic academic or intellectual activity. This may explain the absence of seminal literature within the subject field. As a result, the research tradition within this specific area of mental health service delivery has a relatively short history, with no one dominant researcher or researchers, tradition or seminal studies amongst or across the three traditions identified. |
| Kisely et al. (2017)  Australia | Motivational aftercare planning to better care: Applying the principles of advanced directives and motivational interviewing to discharge planning for people with mental illness | To measure changes in the level of patient input into discharge planning following training staff in the use of MAP | A ward-level controlled before-and-after design comparing one intervention ward with two control wards.  Qualitative interview with individuals discharged from the unit. | Intervention ward: was at Logan Hospital to the south-east of Brisbane (Qld, Australia) that forms part of Metro South Mental Health and Addictions Service.  Two control wards:  (1) another adult general unit at the same hospital (a total of 20 beds and 35 nurses). (2) The hospital within Metro South Mental Health Service, the Princess Alexandra Hospital (28 beds and 30 nurses). | Quantitatively, we initially compared the characteristics of people discharged from the intervention and control wards over the course of the study in terms of sociodemographic features, lengths of stay, and diagnoses. | Following MAP training, the intervention ward improved significantly (e.g. identification of triggers increased from 52 to 94%, v2 = 23.3, d.f.=1, P < 0.001). This did not occur in the control wards.   The qualitative data (n = 20 interviews) showed improvements in participants’ experiences of discharge planning.  MAP increased inpatient input into discharge planning and was valued by participants. The effect on subsequent health service use needs evaluation. |
| Lambert (2019)  UK | Nursing care plans in mental health | To help nurses develop nursing care plans that effectively support the recovery of service users experiencing mental distress. | Literature review | N/A | N/A | The benefits of effective care planning include forming a connection with service users to address their difficulties, improving their ability to self-manage and providing continuity of care. This article has suggested best-practice guidelines and strategies to help nurses improve their clinical skills in using care plans more effectively as part of the nursing process. It is vital that nurses promote meaningful involvement of service users in the care-planning process; a sense of ownership and shared decision making can help to build therapeutic relationships that are essential for recovery. |
| Lodge et al. (2017)  USA | Barriers to Implementing Person-Cantered Recovery Planning in Public Mental Health Organizations in Texas: Results from Nine Focus Groups | To gain varied stakeholder perspectives, three separate focus groups were conducted at each organisation: one focus group with leadership; one focus group with practitioners; and one focus group with peer specialists (i.e., individuals in recovery who are employed to share their experiences and assist others in recovery) | Qualitative study (focus groups) | "Nine focus groups with 71 staff members at the three public mental health provider organisations (1) leadership (i.e., individuals who had been particularly instrumental in implementing PCRP and served in a leadership position at the organisation)  (2) practitioners at the community centres (e.g., case managers, rehabilitation specialists, or qualified mental health practitioners) and treatment team members at the state hospital (e.g., social workers, psychologists, rehabilitation specialists, nurses, and treatment plan coordinators) (3) peer specialists." | Consolidated Framework for Implementation Research (CFIR) was used to present findings from analyses of nine focus groups at three public mental health organisations. | Findings suggest that organisations experienced 12 distinct barriers to PCRP implementation which were categorized into the Consolidated Framework for Implementation Research (CFIR) domains of intervention characteristics, the outer setting, the inner setting, characteristics of individuals, and the implementation process. Half of these 12 barriers fell within the inner setting domain, suggesting that implementation efforts should be flexible and adaptable to organisational culture and context. One-quarter of the barriers fell into the domain of characteristics of individuals involved in the intervention, which further suggests implementation efforts should assess the impact that both staff and consumers have on implementation success. |
| Mancini (2016)  N/R | Mental Health Treatment Planning: A Dis/Empowering Process | To assist social work practitioners in adopting recovery-oriented assessment and treatment planning practices in their day-to-day activities through the establishment of a community of practice called the Co-Occurring Treatment Team (COTT)" | Ethnographic action research (field observations, interviews, and organisational documents (e.g., meeting minutes, policy statements, forms)) | A community mental health centre in a mid-sized metropolitan city in the United States.   12 community mental health caseworkers and two clinical supervisors. | A critical discourse analysis (CDA) was used to explore research question. | The emergence of the recovery model as a guiding vision for mental health treatment systems around the world is a welcome development given the brutality faced by those diagnosed with psychiatric disabilities both historically and currently. However, acknowledgement of the importance of this vision at the policy level is not enough. A warm, respectful, egalitarian working alliance between service user and provider represents one of the main sources of recovery for people diagnosed with psychiatric disabilities. |
| Marston and Weinstein (2013)  UK | Holistic Care Planning for Recovery | N/R | Book Chapter | N/A | N/A | Despite progress in person centred care planning at the policy level, its implementation remains limited in practice, with ongoing concerns about care quality, medical dominance, and insufficient service user involvement. |
| Martinelli et al. (2023)  Italy | Exploring gender impact on collaborative care planning: insights from a community mental health service study in Italy | To investigate: i) differences in the recovery goals of men and women users of a community mental health service in Italy; ii) any differences by gender in recovery over six months using the Mental Health Recovery Star (MHRS) | Prospective cohort study | Service users and staff: 10 women and 15 men completed the MHRS with 19 mental health professionals. | (1) Personal and Social Functioning Scale (FPS) for assessing Personal and social functioning  (2) Nation Outcome Scale (HoNOS) for assessing Psychopathology and social functioning  (3) Monitoring of the Path of Rehabilitation (MPR) for assessing Functional autonomy  (4) Camberwell Assessment of Need (CAN) for assessing Needs for care  (5) Italian version of the MHRS for assessing Personal recovery process and recovery goals | Women tended to choose recovery goals that focused on relationships whereas men tended to focus on work related goals. At follow-up, both men and women showed improvement in their recovery (MHRS) and women were less likely to focus on relationship related goals, perhaps because some had found romantic partners. There were also gains for both men and women in engagement with work related activities. Ratings of functional autonomy (MPR) improved for both men and women, and men also showed improvement in symptoms (BPRS) and functioning (FPS). |
| McHugh and Byrne (2012)  Ireland | The Teamworking Challenges of Care Planning | To examine the teamworking challenges that Community Mental Health Teams (CMHTs) face during the care planning process and how such challenges may be managed | A narrative review | N/A | N/A | Teamworking challenges include the provision of integrated rather than fragmented care, the empowerment of the service user, and development of a distributed model of leadership, responsibility and decision making.  CMHTs face a range of substantial but manageable challenges in attempting to implement recovery-focused care planning. Recommendations include the need to integrate recovery-orientated skills and values into professional training, the need for greater multidisciplinary training opportunities, and the need to evaluate CMHTs based on recovery-orientated criteria. |
| Miller et al. (2017)  USA | Person-centered planning in mental health: A transatlantic collaboration to tackle implementation barriers | Review international efforts to promote PCCP; offer preliminary evidence of PCCP's positive impact on process and recovery outcomes; compare U.S. & Scottish experiences | Conceptual review and implementation report | N/A | N/A | PCCP demonstrably improves recovery outcomes, including medication adherence, engagement, and participation in planning. Its successful implementation has been difficult to achieve in both the US and Scotland. The core barriers are staff attitudes, deficit-focused funding and documentation systems, and unresolved challenges around meaningful outcome measurement. Organisational climate and leadership were also found to matter significantly. Despite operating under very different health systems, both countries faced strikingly similar obstacles, suggesting that the problem is rooted in deeply embedded cultural and systemic forces rather than structural differences. The paper concludes that sustainable progress requires coordinated effort across practice, research, and policy, and that shared transatlantic learning offers a productive path forward. |
| Murphy and Cutts (2009)  UK | Can the introduction of a quality of life tool affect individual professional practice and the quality of care planning in a community mental health team? | The focus of this project was aimed at the outputs of staff in terms of care planning.   The study aimed to explore whether the introduction of the Manchester Short Assessment of Quality of Life (MANSA) gave them insight into alternate working practices and whether as a consequence their care planning activities had changed. | Practice development paper using the clinical note audit and focus group | Community Mental Health Team (a team of mental health care professionals based together in an area outside of a hospital in the local community) specifically: - 3 Social Workers, - 1 Occupational Therapist  - 6 Registered Mental Health Nurses | N/A | The use of MANSA with care planning proved enlightening and affirming to practitioners and engaged them in considering value-based practice (recovery-orientated) from the perspective of the client. It was clear that subtle changes to care planning emerged, and the goal of giving responsibility for self-determining issues was addressed. |
| O'Donohue et al. (2023)  Australia | Mental health consumer perspectives of a person-centred multidisciplinary care planning meeting on a rehabilitation inpatient unit | To evaluate the consumer perspective of a person-centred multidisciplinary care planning meeting (referred to in this article as personal planning and review (PP&R) meetings | Qualitative study: ethnography (field notes, observations of the PP&R meeting), semi-structured interviews) | Interviews: only with consumers (n=8)  Observations of the PP&R meeting | After the participant responded using the Visual Analogue Scale (VAS) they were asked follow-up questions such as ‘what made you choose that response?’, ‘can you provide me with any examples of why you have chosen that response?’ | Findings showed that consumers' experiences of the care planning meetings were positive. Themes included; ‘It's about you’, ‘Making decisions and expressing opinions’, ‘Staff involvement in care planning’ and ‘Supporting consumer recovery’. These findings add the consumer perspective to the existing evidence base and support the implementation of person-­ centred multidisciplinary care planning meetings in inpatient mental health settings. |
| Palmer et al. (2014)  Australia | Written plans: an overlooked mechanism to develop recovery-oriented primary care for depression? | To report on the views of a large cohort of primary care users with various levels of severity of depressive symptoms about the importance and bene ts of having access to a written plan to recover from depression. | Mixed-method study: longitudinal study + follow up telephone interviews | 789 participants | Quantitative: the Centre for Epidemiological Studies– Depression Scale (CES-D) and the Patient Health Questionnaire (PHQ)assessing depressive symptoms | The benefits of having a written plan to recover from depression, as outlined by the participants, were analysed using Leximancer software. The findings provide insights into how written plans may be an important mechanism for implementing a recovery-oriented primary mental health care system. |
| Pithara et al. (2020)  UK | Implementing a Digital Tool to Support Shared Care Planning in Community-Based Mental Health Services: Qualitative Evaluation | To examine mental health care providers’ views of and experiences with the CPT during the pilot implementation phase and identify factors influencing its implementation | Qualitative study (in-depth telephone interviews) | 20 staff | N/A | The innovative mobile digital care pathway tool was thought to facilitate coproduced recovery-focused care planning, a policy and organizational as well as professional priority. Internet connectivity issues, system interoperability, and access to service users’ health records affected use of the tool during mobile working. |
| Reid et al. (2018)  Australia | Collaboration as a process and an outcome: Consumer experiences of collaborating with nurses in care planning in an acute inpatient mental health unit | To explores inpatient mental health consumer perceptions of how collaborative care planning with mental health nurses impacts personal recovery | Qualitative study: semi-structure interviews | Consumers close to discharge from one unit in Sydney, Australia. | N/A | Findings are presented under four organizing themes: the process of collaborating, the purpose of collaborating, the nurse as collaborator and the role of collaboration in wider care and recovery. Consumers highlighted the importance of the process of developing their care plan with a nurse as being as helpful for recovery as the goals and strategies themselves. |
| Rinaldi and Watkeys (2014)  UK | Do our current approaches to care planning and the CPA enhance the experience and outcomes of a person’s recovery? | To describe aspects of the current situation with regard to the effectiveness of care planning in supporting a person’s recovery | Conceptual paper | N/A | N/A | Within recovery focused services a care plan becomes the driving force, or action plan, behind a person’s recovery journey and is focused on their individual needs, strengths, aspirations and personal goals. If involving people directly in the development of their care plan is critical to creating better outcomes then supporting self-management, shared decision making and coproduction all underpin the care planning process. Based on the evidence of people’s experience of care plans and the care planning process it is time to seriously debate our current conceptualisation and approach to care planning and the future of the CPA. |
| Rio et al. (2021)  Australia | The impact of workplace culture on the accountability of mental health nurses to involve consumers in care planning: A focused ethnography | To explore the impact of workplace culture on mental health nurses’ capacity to involve consumers in care planning and to work accountably | Qualitative: focused ethnography involving in-depth semi-structured interviews and non-participant observation | Interview participants: 12 nurses (10 registered nurses and 2 enrolled nurses, all qualified in mental health).  Observed professionals: 23 additional health professionals during nonparticipant observation. | N/A | The main finding was that workplace culture constrained recovery oriented, consumer involved care planning. In practice, a culture dominated by reduced length of stay, crisis work, safety, and risk management meant that care planning was often reactive, superficial, and not meaningfully collaborative. Care plans became devalued, nurses had limited voice in team discussions, and consumer involvement was not prioritised. These are strongly supported by the findings and reinforced in the discussion. |
| Rylance and Graham (2014)  UK | Does the practice of care planning live up to the theory for mental health nursing students? | Aim/objective: To examine the perceptions of mental health nursing students and their observations of care planning in theory versus practice following evidence-based teaching sessions. The study explores the "theory-practice gap" in person-centred care | Qualitative study | Six third-year mental health nursing students participated. They were recruited after taught sessions on care planning. Practice settings included inpatient and specialist community placements. | N/A | The paper’s core finding is that the recovery-focused, collaborative care planning taught in university was often not reflected in clinical practice. Students perceived care plans in practice as often: - not person-centred, - not genuinely collaborative, - problem-focused rather than goal-focused, - shaped by organisational pressures, - constrained by risk management, - and reduced to a bureaucratic or tick-box exercise. The paper states four main findings categories: 1. care planning custom and practice 2. collaboration 3. organisational culture 4. student assumptions about mentors |
| Shue et al. (2023)  USA | Exploring Factors Impacting the Implementation of Recovery-Oriented Treatment Planning on Acute Inpatient Mental Health Units | To systematically examine factors affecting the implementation of recovery-oriented inpatient treatment planning in a large and diverse national sample of VHA inpatient mental health units | Qualitative study | 34 VHA inpatient mental health units in 13 sites: - 8 high-scoring sites - 5 low-scoring sites | Recovery-Oriented Acute Inpatient (RAIN) Scale | The study found that implementation of recovery-oriented inpatient treatment planning was shaped by both common cross-site patterns and key differentiators between high- and low-scoring units. Across sites, there was a dominant acute care philosophy focused on stabilization, safety, and medication management. High-scoring sites differed from low-scoring sites in more consistently: - involving veterans meaningfully in treatment planning, - eliciting recovery goals beyond short-term clinical goals, - valuing veteran input in medication decisions, - aligning group programming with recovery, - and involving family/significant others in treatment discussions rather than only for safety/discharge purposes. |
| Simpson et al. (2016)  UK | Recovery-focused care planning and coordination in England and Wales: a cross-national mixed methods comparative case study | To identify and describe factors associated with collaborative, personalised, recovery-focused care planning and coordination in community mental health services, and to examine stakeholders’ views and experiences across England and Wales | Mixed-methods study | The study included: - service users: n = 448 survey respondents - care coordinators: n = 201 survey respondents - interview participants: service providers, service users, and carers n = 117 - care plan reviews: n = 33 - sites: six provider sites across England and Wales. | - Perceptions of recovery-oriented practice, empowerment, and therapeutic relationship - Recovery Self-Assessment Scale (RSA) for recovery-oriented practices - Scale to Assess the Therapeutic Relationship (STAR-P) - Empowerment Scale (ES). | The main finding was that, despite policy requirements for collaborative, personalised, recovery-focused care planning, practice was often administratively burdensome, weakly collaborative, inconsistently recovery-focused, and insufficiently used in everyday care. Care plans were rarely consulted after completion; carers were variably involved; risk assessment was central for staff but rarely openly discussed with service users; and therapeutic relationships were more strongly valued than the written care plan itself. The paper also found: - significant site differences in therapeutic relationship scores; - no substantive site differences in overall recovery-oriented practice or empowerment scores; - strong positive association between recovery-oriented practice ratings and therapeutic relationship ratings. |
| Tondora et al. (2012)  USA | The top ten concerns about person-centred care planning in mental health systems | To describe the top ten concerns commonly raised by practitioners about implementing person-centred care planning in mental health systems and to address these concerns within the context of recovery-oriented care | A conceptual paper | N/A | N/A | The overall argument is that person-centred care planning is a core component of recovery-oriented mental healthcare and that common objections about risk, liability, documentation, time, reimbursement, patient capacity, and professional authority can be addressed without abandoning rigorous care planning. |
| Wyder et al. (2018)  Australia | The language we use – the effect of writing mental health care plans in the first person | To describe the impact of Motivational Aftercare Planning (MAP) | Mixed-methods study | The data consisted of 110 anonymised recovery plans: - 55 plans pre-intervention - 55 plans post-intervention. The intervention was trialled in a 20-bed acute adult psychiatric ward staffed by 37 nurses at a major teaching hospital in Brisbane. | N/A | The main finding was that the intervention produced a clear shift in the thematic content of recovery plans. Plans completed before the intervention were more clinician-focused, with emphasis on: - decreasing symptoms, - acceptance of illness, - clinical stability, - risk management, - treatment compliance.  Plans completed after the intervention were more person-focused, with emphasis on: - general wellness, - meaningful activities, - community life, - social roles, - social connection. |

**References**

Berger, J.L., 2006. Incorporation of the Tidal Model into the interdisciplinary plan of care--a program quality improvement project. J Psychiatr Ment Health Nurs 13 (4), 464–467.

Borkman, T.J., 1998. Is Recovery Planning Any Different from Treatment Planning? Journal of Substance Abuse Treatment 15 (1), 37–42.

Branjerdporn, G., Healey, L., Hudson, C., 2023. Understanding care plans in a psychiatric mother-baby unit. J Reprod Infant Psychol 41 (5), 566–581.

Dadich, A., Fisher, K.R., Muir, K., 2013. How can non-clinical case management complement clinical support for people with chronic mental illness residing in the community? Psychology, Health & Medicine 18 (4), 482–489.

Ebihara, M., Tamura, T., Masukawa, N., Omiya, T., Ando, K., 2026. Exploring the Process of Professional Role Redefinition Towards Recovery-Oriented Care Through Joint Crisis Plans in Japan: A Qualitative Study Using the Modified Grounded Theory Approach. In: Healthcare. pp. 1003.

Hipfner, C.M., Bennett, L., Gettle, D., New, C., Howell, S., 2017. Teaching recovery principles with concept map care planning. The Journal of Mental Health Training, Education and Practice 12 (5), 292–299.

John, T., 2022. Service user involvement in recovery-oriented care planning: a realist synthesis. In: Centre for Health Services Studies. University of Kent, England.

Jones, A., Hannigan, B., Coffey, M., Simpson, A., 2018. Traditions of research in community mental health care planning and care coordination: A systematic meta-narrative review of the literature. PLOS ONE 13 (6), e0198427.

Kisely, S., Wyder, M., Dietrich, J., Robinson, G., Siskind, D., Crompton, D., 2017. Motivational aftercare planning to better care: Applying the principles of advanced directives and motivational interviewing to discharge planning for people with mental illness. International Journal of Mental Health Nursing 26 (1), 41–48.

Lambert, N., 2019. Nursing care plans in mental health. Mental Health Practice 28 (5).

Lodge, A.C., Kaufman, L., Stevens Manser, S., 2017. Barriers to Implementing Person-Centered Recovery Planning in Public Mental Health Organizations in Texas: Results from Nine Focus Groups. Adm Policy Ment Health 44 (3), 413–429.

Mancini, M.A., 2016. Mental health treatment planning: A dis/empowering process. In: O'Reilly, M., Lester, J.N. (Eds.), The Palgrave Handbook of Adult Mental Health: Discourse and Conversation Studies. Springer, pp. 352–370.

Marston, D., Weinstein, J., 2013. Holistic Care Planning for Recovery. In: Hall, A., Wren, M., Kirby, S. (Eds.), Care planning in mental health: Promoting recovery. John Wiley & Sons.

Martinelli, A., Bonetto, C., Pozzan, T., Procura, E., Cristofalo, D., Ruggeri, M., Killaspy, H., 2023. Exploring gender impact on collaborative care planning: insights from a community mental health service study in Italy. BMC Psychiatry 23 (1), 834.

McHugh, P., Byrne, M., 2012. The Teamworking Challenges of Care Planning. Ir J Psychol Med 29 (3), 185–189.

Miller, E., Stanhope, V., Restrepo-Toro, M., Tondora, J., 2017. Person-centered planning in mental health: A transatlantic collaboration to tackle implementation barriers. Am J Psychiatr Rehabil 20 (3), 251–267.

Murphy, N., Cutts, H., 2009. Can the introduction of a quality of life tool affect individual professional practice and the quality of care planning in a community mental health team? J Psychiatr Ment Health Nurs 16 (10), 941–946.

O'Donohue, E., Aguey-Zinsou, M., Yule, E., Fairhurst, I., Debaets, M., 2023. Mental health consumer perspectives of a person-centred multidisciplinary care planning meeting on a rehabilitation inpatient unit. Int J Ment Health Nurs 32 (6), 1701–1712.

Palmer, V.J., Johnson, C.L., Furler, J.S., Densley, K., Potiriadis, M., Gunn, J.M., 2014. Written plans: an overlooked mechanism to develop recovery-oriented primary care for depression? Australian journal of primary health 20 (3), 241–249.

Pithara, C., Farr, M., Sullivan, S.A., Edwards, H.B., Hall, W., Gadd, C., Walker, J., Hebden, N., Horwood, J., 2020. Implementing a Digital Tool to Support Shared Care Planning in Community-Based Mental Health Services: Qualitative Evaluation. J Med Internet Res 22 (3), e14868.

Reid, R., Escott, P., Isobel, S., 2018. Collaboration as a process and an outcome: Consumer experiences of collaborating with nurses in care planning in an acute inpatient mental health unit. Int J Ment Health Nurs 27 (4), 1204–1211.

Rinaldi, M., Watkeys, F., 2014. Do our current approaches to care planning and the CPA enhance the experience and outcomes of a person's recovery? The Journal of mental health training, education and practice 9 (1), 26–34.

Rio, J.H.M., Fuller, J., Taylor, K., Muir-Cochrane, E., 2021. The impact of workplace culture on the accountability of mental health nurses to involve consumers in care planning: A focused ethnography. Nurs Health Sci 23 (1), 255–262.

Rylance, R., Graham, P., 2014. Does the practice of care planning live up to the theory for mental health nursing students? Mental Health Practice 18 (2).

Shue, S.A., Traylor, M., Kukla, M., Salyers, M.P., Rollins, A.L., Henry, N., Eliacin, J., Garabrant, J., McGuire, A.B., 2023. Exploring Factors Impacting the Implementation of Recovery-Oriented Treatment Planning on Acute Inpatient Mental Health Units. Adm Policy Ment Health 50 (2), 283–295.

Simpson, A., Hannigan, B., Coffey, M., Barlow, S., Cohen, R., Jones, A., Všetečková, J., Faulkner, A., Thornton, A., Cartwright, M., 2016. Recovery-focused care planning and coordination in England and Wales: a cross-national mixed methods comparative case study. BMC Psychiatry 16, 147.

Tondora, J., Miller, R., Davidson, L., 2012. The top ten concerns about person-centered care planning in mental health systems. International Journal of Person Centered Medicine 2 (3), 410–420.

Wyder, M., Kisely, S., Meurk, C., Dietrich, J., Fawcett, T., Siskind, D., Robinson, G., Crompton, D., 2018. The language we use–the effect of writing mental health care plans in the first person. Australasian Psychiatry 26 (5), 496–502.
